# The Chicago Consensus on Sustainable Food Systems Science

**DOI:** 10.3389/fnut.2017.00074

**Published:** 2018-04-25

**Authors:** Adam Drewnowski, The Ecosystem Inception Team, Adam Drewnowski

**Affiliations:** University of Washington, Seattle, WA, United States

**Keywords:** food systems, nutrition, sustainability, cost, environment

## Abstract

As participants at the Ecosystem Inception Meeting convened by the Global Dairy Platform and held in Chicago in June 2016, we have identified some concepts as central to the study of food systems science. Following the definition developed by the Food and Agriculture Organization for sustainable diets, the food supply needs to provide foods that are healthy and safe, affordable, culturally acceptable, and with low impact on the environment. Therefore, the four main domains of sustainable food systems science can be described as health, economics, society, and the environment. Food systems science needs to embrace and engage with all relevant allied disciplines that may include environmental health sciences, epidemiology, geography, history, sociology, anthropology, business, and political science. Research and training in food systems science, both domestic and international, would benefit from a set of competencies, from more extensive research networks, and from more public–private engagement. This document builds on major advances in the area of food system research, training, and practice, already achieved by individuals, institutions, foundations, and local and national governments.

## Elements of Sustainable Food Systems Science

The operations of modern agricultural and food systems span the continuum from food production and processing to food consumption, nutrition security, and population health. The chain of food production ranges from agriculture and livestock to commodity trades, food processing, distribution, and retail. On the consumption side are economic, cultural, and behavioral factors, ranging from the pricing and marketing of foods to individual and group food purchases and food consumption patterns. Food systems, both local and global, can be profoundly influenced by the perturbations in the food supply and by changing consumer demand.

The operations of the global food system can have a profound impact on economies and trade, as well as on political stability at local, regional, and global scales. The food system can affect, if not shape, current and future food and nutrition security and global population health. The food system can be disrupted by social, economic, and political events as well as by environmental factors that may involve depletion of natural resources, global warming, and climate change. Conversely, economic development, as manifested by the nutrition transition, can affect food choices and eating habits on a population scale.

The four domains of sustainable food systems science can also be conceptualized as health, economics, society, and environment. Research and training in the new discipline, with population health at its core, are by definition, interdisciplinary (1). Consistent with the principles of the Giessen Declaration (2), the “new” nutrition science is ready to embrace and engage with diverse allied disciplines that may include environmental health sciences, epidemiology, geography, history, sociology, anthropology, business, and political science. Research and training in food systems science would benefit from a set of curriculum competencies, from more extensive research networks, and from more public-private engagement.

## The Ecosystem Inception Meeting

The goal of the 2016 Chicago Ecosystem Inception workshop was to create an international network of researchers working on food system issues from the perspectives of nutrition, epidemiology, economics, social sciences, agriculture, climate and the environment. Systematic working collaborations make it easier for individual scientists to become a part of larger research networks. Larger networks facilitate the flow of resources: funders generally look for an international scope and for broad impact of proposed research.

The workshop process was facilitated by the Global Dairy Platform. Providing an open forum for an exchange of ideas and methodologies was the first step toward creating a network of engaged actors and stakeholders. The need for more interdisciplinary studies can sometimes run counter to academic traditions. Researchers in academic institutions are often segregated into departmental silos. The workshop goal was to allow participants to engage with their own stakeholders, effectively functioning as a research ecosystem, expanding its reach to academia, governments, industry, and the civil society. The workings of the food system are of interest not only to agro food industries but also to banks, insurance companies, and foundations and NGOs.

A better understanding of how food systems operate would be valuable to governmental agencies, public health jurisdictions, and the private sector (3, 4). Aligning the food systems research agenda with the UN Sustainable Development Goals will require active engagement of multiple stakeholders and multi-sector actors from academia, agro food industries and smallholders, national and international civil society NGOs, and local and national governments (3, 5). Nourishing the global population, estimated at 9 billion by year 2050, will test food system sustainability and resilience.

The workshop goal was to frame a shared research agenda and to develop future research priorities and goals. Working toward a preliminary consensus, the group began by developing some working definitions of food systems science (Box 1). Workshop participants shared their collective expertise to define key issues and common themes and identified some key challenges. The group identified the need to build a conceptual framework, examine the existing evidence base, and develop new analytical models. There was also an identified need to reflect on the scope and the quality of some of the existing scientific evidence and on the limitations of some of the current research.

Box 1 **Key definitions of sustainability, food systems, and food and nutrition security.**

**Table:** 

Terms	Definition
Sustainability	Sustainability implies a state where the needs of the present and local population can be met without diminishing the ability of future generations or populations in other locations to meet their needs or without causing harm to environment and natural assets (8) “The ability to continue social, environmental and economic behavior indefinitely” (9, 10)

Sustainable development	“Development which meets the needs of current generations without compromising the ability of future generations to meet their own needs.” It contains within it two key concepts: • The essential needs of the world’s poor, to which overriding priority should be given. • Limitations imposed by both technology and society on the environment’s ability to meet present and future needs (11)

Food and Nutrition Security	“All people at all times have physical, social and economic access to food, which is safe and consumed in sufficient quantity and quality to meet their dietary needs and food preferences, and is supported by an environment of adequate sanitation, health services and care, allowing for a healthy and active life” (3)

Sustainable diet	“Sustainable diets are those diets with low environmental impacts that contribute to food and nutrition security and to healthy life for present and future generations. Sustainable diets are protective and respectful of biodiversity and ecosystems, culturally acceptable, accessible, economically fair and affordable, nutritionally adequate, safe, and healthy, while optimizing natural and human resources” (4)

Food system	“An interconnected web of activities, resources and people that extends across all domains involved in providing human nourishment and sustaining health, including production, processing, packaging, distribution, marketing, consumption and disposal of food” (12)

Sustainable food system	“Characterized by agricultural productivity, diversity of food supply, the economic accessibility of food to consumers, use of natural resources for agriculture, and urbanization of the population living from the system” (13)

Following the academic model of research, service, and teaching, the group recognized the need to train a new cadre of food system professionals. Curriculum and course development were among the topics discussed. Some building blocks for sustainable food systems research and training were identified.

## The Four Domains of Sustainable Food Systems

Following the definition developed by the Food and Agriculture Organization (4), sustainable diets need to be healthy and safe, affordable, culturally acceptable, and with low impact on the environment. Based on the definitions above, the four main domains of sustainable food systems science can also be conceptualized as health, economics, society, and the environment. It is important to note that the environmental impact of foods is only one of the four dimensions of sustainability.

While there was agreement that food production systems need to be sustainable at all time scales, the definition of food system “sustainability” remains both elusive and complex. Sustainable food systems were defined by FAO as those that managed production and biodiversity, while sparing human and natural resources and developing resilience to a wide range of stresses and shocks (6).

The World Economic Forum has placed more emphasis on food system economics. Sustainable food systems were those that delivered healthy foods in a profitable manner and were demographically and culturally inclusive, as well as sparing of the environment. This approach, more concerned with the economics and society dimensions, incorporated fair labor practices; the creation of markets; the development of business infrastructure; the roles of tariffs and trade; and the impact of labor, agricultural, and environmental policies on food value chains.

The study of food systems allows for an integration of public health nutrition with key concepts in sociology and political science.

## Models, Metrics, and Measures

Each of the four principal domains of sustainability has its own models, metrics, and measures. The adequacy and nutrient density of food patterns have been measured in terms of both nutrients and energy (calories). Nutrient profiling models can separate foods that are energy dense from those that are nutrient rich. Economic metrics include food poverty and food affordability, the latter measured in terms of dietary calories and nutrients per unit cost. Cultural acceptance of foods by region can be measured in terms of frequency of consumption by population subgroups. The environmental costs of farming and food production have been measured in terms of land use, water use and quality, and greenhouse gas emissions (GHGEs), expressed in carbon dioxide equivalents per kilogram of food. Health impacts have been measured using summary measures of population health such as disability-adjusted life years.

All these models and measures require extensive data inputs at multiple levels, from local to national to global. The required data inputs can range from agricultural production and weather events to dietary intakes data and health outcomes.

The health dimension addresses the quality and the safety of the food supply. Measures are needed to determine whether the foods produced are nutrient rich, safe, accessible, and appealing. The health dimension is also concerned with food choices and consumption patterns and with the impact of food patterns on nutritional status, disease risks, and population health. Analyses of the impact of food systems on health outcomes will require quantitative and qualitative data on dietary intake assessment, food-related attitudes and beliefs, and measures of population health.

The economics dimension addresses the economic aspects of food supply and food demand, incorporating diverse historical, geopolitical, and business perspectives. Food prices are an important aspect of food choice and not only in low- and middle-income countries (LMICs). An important element here is power relations that make food systems equitable and fair, with implications for food and social justice. While the research focus has been on the corporations, a vast amount of food is produced by local smallholders, with a number of associated social and environmental issues. Among data needs are local and regional food prices, as well as data on economic equity and food and economic policies by region.

The societal dimension recognizes that cultural, social, and religious factors can drive food choices. The global nutrition transition can be viewed as the combined outcome of economics and culture. The study of local to global food systems should consider social structures, regional differences, and cultural norms (7). In particular, the role of communities striving to develop and establish alternative food systems should be considered. The importance of the societal dimension opens the door to qualitative research in nutrition, focused on food-related attitudes and behaviors.

Finally, the environmental dimension addresses the impact of the food system operations on land, water, and energy use, at local and at global scales. This dimension includes the concept of food system resilience to extreme weather, climate change, resource degradation, and other forms of environmental stress. Included here is the “one health” approach, which addresses the risk to humans of zoonotic diseases, antibiotic resistance, and other influences of the food production environment on human health.

More data are needed to assess food systems resilience to gradual challenges or sudden shocks, whether caused by global warming or by societal events. Modeling can show whether global economic pressures exacerbated by climate change will affect the global food supply and shape future diets. Studies have already shown that price spikes and food scarcity have led to deprivation, hunger, and even political unrest.

## A Continuing Need for High-Quality Data

Food system sustainability criteria are increasingly being factored into discussions of agriculture, food processing, food patterns, dietary guidelines, nutrition and health, and the environment. There are growing concerns that global food production, increasingly reliant on intensified agriculture, can result in the depletion or degradation of natural resources, including land, water, and energy resources. These risks to sustainability can be exacerbated by global warming and climate change. However, incipient research in these areas faces multiple challenges.

First, the lack of appropriate data has blocked the development of models that aimed to link sustainable diets with current or future nutrition and health outcomes. The consensus group identified some major gaps in data inputs. Data aggregation was one problem. Often, data were collected at many different interacting scales, from regional to national. Local data on the environmental impact of animals and crops, including GHGEs or land or water use, were particularly hard to obtain. Studies on the environmental impact of commodities, food groups, or specific diets rarely had access to data that were specific to a given time or geographic location.

One of the key issues in the study of food systems is that environmental resources and burdens can be highly local. Water use, soil depletion, and eutrophication are all context dependent.

Lifecycle analysis (LCA) of foods, a very rough summary measure of environmental impact, is very much context dependent; data from the European Union do not necessarily apply to Australia or the United States. LCA impact will change if the farming systems and the food chain would become more resource efficient. While LCA can be useful at the production level, other measures may be required to assess the environmental aspect of food processing and packaging and to assess the consumer waste. The optimization of food value chains needs LCAs (or similar methods) to account for consecutive steps in the food production chain from production to waste.

At this time, food product-level LCA data for the US are extremely limited. Data from France are limited to GHGEs and eutrophication. Data on water use are only gradually becoming available. New models on the nutrition and environmental aspects of plant-source versus animal-source foods will be able to make use of more detailed local data, better reflecting local production conditions. We need a better understanding of how food system activities affect the environment and climate change and how climate is likely to disrupt food systems at the local and national levels. There are concerns that excess pesticide and fertilizer use and reliance on monocultures have led to diets that exert an overly high environmental toll.

Second, there was a dearth of verifiable models. Linking agricultural production with nutritional status and health outcomes has been problematic. Although agricultural production data are usually collected at some level of geographic aggregation, consumption and health data, when available, are invariably collected at the household or individual level. Agricultural production data by area can be expressed per capita and can be adjusted for waste. However, such methods are merely proxies for dietary intake assessment, which has its own problems and can be biased by a host of sociodemographic variables.

Linking per capita agricultural production data to individual food consumption has been difficult; sociodemographic surveys of food consumption are rare, especially in LMICs. The joining of national food security data with nutritional status and health outcomes was equally challenging: there are many factors in the pathway, ranging from hygiene and sanitation to empowerment of women and maternal and child health. Although many existing models have focused on subsistence farming outcomes, rapid urbanization in LMIC needs urgently to be taken into account.

Data on prevailing food prices at the national level were rarely available, especially for LMICs. Data on nutrient composition of foods and dietary patterns could be scarce, as were data on health outcomes. Data on the socioeconomic drivers of food choice were limited: consumption patterns and health outcomes are not evenly distributed across social strata.

As a result, the group noted that existing studies on sustainable foods and sustainable diets have, thus far, produced only partial and fragmentary answers. The lack of empirical data inputs with which to build and test models was one challenge. The lack of models that could be subjected to testing and verification was another. There was definitely a need to advance the quality of the debate on food systems sustainability and to improve the understanding of potential trade-offs.

## The Integrated Food Systems Approach

While there is a need for more data inputs and models, there is also a need to place the current data and concepts into a broader and more health oriented perspective. The interdisciplinary nature of food systems research calls for active engagement by researchers from diverse disciplines and multiple geographic areas.

Future studies on sustainable food systems and sustainable diets must address a wide range of interconnected, social, economic, and environmental factors. For example, global warming and catastrophic weather events affect crop yields and livestock, affect food transport and storage, cause spikes and volatility in food prices, and can lead to social and economic unrest. Antibiotic resistance and zoonoses can also originate from animal production systems.

Multidisciplinary research on the economics of food choice behavior and its nutrition and health outcomes would be useful. Sweeping changes in food system practices have been proposed as ways to mitigate the environmental impact of food production. Among the suggested remedies were plant-based diets and the removal of livestock, especially beef and dairy cattle, from the food supply. Such arguments, often based on incomplete data on the environmental impact of food production, need to take multiple other factors into account.

Sweeping changes in consumer behavior may not be socially or economically viable. The removal of livestock or dairy from the food supply, in particular, is not a realistic option. Livestock is a key to the economy of many countries and is especially important for smallholder farmers in LMIC. As permanent pasture makes up the bulk of the agricultural land on the planet, livestock are a major source of food and livelihoods.

There are also trade-offs to be made between the nutritional and the environmental domains of sustainability. The key nutrient deficiencies in LMIC populations consuming high carbohydrate, mostly plant-based, diets are in iron, calcium, zinc, and vitamin B12. Animal products supply vitamin B12 and the consumption of animal source foods may contribute to fight undernutrition. Although some Western societies move to limit animal foods, LMICs still depend on meat, poultry, and fish and on dairy as the chief sources of high-quality protein. Food system research will need to provide insight into policies and suggest realistic multi-sector interventions.

Trade-offs between nutrition and the environment may be the hardest to address. Many current discussions on sustainable diets appear to be driven by environmental considerations, without a full consideration of the other three domains: nutrition, economics, and culture. The key challenge in livestock production is to improve efficiency while reducing environmental impact. Studies need to recognize the value of water recharge and environmental services and the importance of converting non-consumable agricultural products into consumable and highly nutritious foods. Existing models need to take context-specific uses of grazing lands, open space, and water recharge areas into account.

There is a need for more holistic studies on modeling the impact of climate change on food production (including, but not limited to, agricultural yields); economic modeling of prices and their impact on food purchases, food security, and health. Additional models will need to be developed to test the resilience of food systems to political and weather scenarios that could result in a catastrophic breadbasket failure.

## From Food Systems to Public Policy

Feeding the world in the face of population growth, increasing wealth, but also rising poverty, and climate change poses not only new challenges to agro food industries but also new opportunities. Among rising challenges are the feeding of emerging megacities in Africa, Asia, and Latin America. The transformation of urban food systems has been extremely rapid; however, they are fast becoming centers of malnutrition, obesity, and diabetes.

Establishing convincing links between food systems and nutrition and health outcomes will be a critical component of this work. Models will need to predict the likely impact of future food systems on food and nutrition security and on different forms of malnutrition, from wasting and stunting to higher risk of non-communicable disease, including obesity and diabetes. Private–public partnerships will be required, and the global agri food industries will have a major role to play.

Food patterns high in animal products have been criticized for being both unhealthy and environmentally unsustainable. One concern has been that intensive agricultural production makes use of non-renewable land, water, and energy resources and contributes to global warming, but this mainly considers Western, intensive animal husbandry systems. Meat and dairy production does require more land and water resources than other foods and contributes significantly to GHGEs. The question is whether the environmental cost is offset by the products higher nutritional value and how far the current environmental impact of animal source food production can be reduced by improving efficiency while increasing production.

Currently, cereal and oilseed crops, rather than meat or dairy, account for most of the calories in the global food supply. Corn, wheat, rice, soy, and sugar cane are all current staples that yield inexpensive dietary energy and provide plant-based fat, refined carbohydrates, and protein. Other than soy none of these crops are significant sources of protein, and the quality of the protein is low relative to animal products. Plant-based diets in LMIC are inadequate sources of micronutrients and vitamins (particularly vitamin B12).

At this time, grains, sugars, and vegetable fats are generally cheaper per calorie than are some nutrient-rich foods. Although carbohydrates and oils continue to be essential elements of balanced nutrition, there is pressure and, from a nutritional viewpoint, a need in LMIC for more animal-based diets.

The question whether healthy diets are also good for the planet would benefit from a more extended debate. Consumers want their foods to be healthy, safe, affordable, culturally acceptable, and appealing and are becoming increasingly sensitive to environmental concerns. It may turn out that no single diet or food pattern fits all of the criteria; some compromises may need to be made. One research question is whether increased population demand for calories will intensify the pressure to increase the yields of low-cost, energy-dense crops. Those plant foods have been linked to higher rates of obesity and non-communicable diseases worldwide. However, societal aspirations are often tied with food availability and drive food-seeking behaviors.

There are limited data on the drivers of food choice and the potential points for dietary intervention. These studies will require a focus on behavior, nutrition, and culture and ethics. For example, the advice to bypass animals and consume plant foods directly is liable to meet with consumer resistance. Plant-based diets are already associated with micronutrient deficiencies in the LMIC. Fueled by the nutrition transition in the LMIC, diet patterns have changed dramatically over the past few decades and vary enormously between cultures. There is a need to better understand the drivers of food choice, their dynamics, and interrelationships with consumer behavior. There is a further need to explore perceptions, attitudes, and motivations and their role in formulation of public policies.

## Conclusion

Measuring diet quality in relation to environmental impact and health outcomes are very much a part of the ongoing sustainability debate. To enable capacity building in food systems research, we need both curricula and research infrastructures. Training will produce a new cadre of food systems professionals. Shared research methodologies are needed to standardize data, tools, methods to allow data exchange and food systems modeling.

The existing food systems need to assure global food security in the face of population growth and climate change. Sustainable nutrition needs to balance nutrient requirements with energy needs. The goals are to reduce hunger, undernutrition, and stunting while avoiding the risk of obesity and other diet-related NCDs.

## Contributor’s Information

### The Ecosystem Inception Team

**Adam Drewnowski**, Center for Public Health and Nutrition, University of Washington, Seattle, WA, United States; **Arie Havelaar**, Department of Animal Sciences, Institute for Sustainable Food Systems and Emerging Pathogens Institute, University of Florida, Gainesville, FL, United States; **Carlos Sere**, Bioversity’s International, CGIAR, Italy; **Charlotte de Fraiture**, Water Science Engineering Department, UNESCO-IHE, Netherlands; **Frank Mitloehner**, Department of Animal Science, University of California, Davis, CA, United States; **Henning Steinfeld**, Livestock, Environment and Development Initiative (LEAD), FAO, Italy. **Hugo Melgar-Quinonez**, McGill Institute for Global Food Security, Quebec, QC, Canada; **John Ingram**, Environmental Change Institute (ECI), University of Oxford, Oxford, United Kingdom; **Martin Heller**, Center for Sustainable Systems, University of Michigan, Ann Arbor, MI, United States; **Pieter van’t Veer**, Division of Human Nutrition Wageningen University, Netherlands; **Roger Clemens**, Regulatory Sciences Program, University of Southern California, Los Angeles, CA, United States; **Shenggen Fan**, International Food Policy Research Institute, Washington, DC, United States; **Terry Marsden**, Sustainable Places Research Institute, Cardiff University, Cardiff, United Kingdom; **Timothy Griffin**, Friedman School of Nutrition Science and Policy, Tufts University, Boston, MA, United States.

## Author Contributions

All authors listed have made a substantial, direct, and intellectual contribution to the work and approved it for publication.

## Conflict of Interest Statement

AD has received grants, honoraria, and consulting fees from numerous food, beverage, and ingredient companies and other commercial and non-profit entities with an interest in the nutrient density of foods. The University of Washington receives research funding from public and private sectors.
